# Plasmid-Mediated AmpC (pAmpC) Genotypes Among Uropathogenic Escherichia coli: A Hospital-Based Study From Western Uttar Pradesh

**DOI:** 10.7759/cureus.41551

**Published:** 2023-07-08

**Authors:** Ismat Rehana, Anita Pandey, Peetam Singh

**Affiliations:** 1 Microbiology, Subharti Medical College, Meerut, IND

**Keywords:** ampc beta (β)-lactamases, cefoxitin, cit, ampc genes, pcr

## Abstract

Introduction

Resistance due to AmpC and extended-spectrum beta (β)-lactamases (ESBLs) in *Escherichia coli* is an emerging problem worldwide. AmpC enzymes are a subclass of β-lactamases that have a capacity to hydrolyze and deactivate a large range of β-lactam antibiotics, particularly cephalosporins, penicillins, and monobactams, although frequently being susceptible to carbapenems and fourth-generation cephalosporins. The prevalence of plasmid-mediated AmpC (pAmpC) genotypes in uropathogenic *E. coli *isolates were looked at a tertiary care teaching hospital of Western Uttar Pradesh.

Materials and methods

A total of 312 non-repeat clinical *E. coli *isolates among patients presented with urinary tract infections (UTIs) were investigated by standard microbiological methods. Isolates were screened for the presence of ampC using a cefoxitin (30 µg) disc and confirmed using an inhibitor-based assay. Using multiplex polymerase chain reaction (PCR), six AmpC genotypes, namely, CIT, DHA, EBC, ACC, FOX, and MOX, were genotypically identified.

Results

A total of 152 (48.72%) uropathogenic *E. coli* isolates tested positive on the cefoxitin screening. Out of which, AmpC production was confirmed in 118/152 (77.63%) using a phenotypic method. In particular, the pAmpC gene was found in 56/152 (36.84%) isolates. CIT was the most common gene detected in this geographical area (57.14 %). Multiple genes, i.e., CIT and FOX, were also detected in 14.29% of the isolates.

Conclusion

Identifying AmpC producers is important in routine microbiology laboratory as they are a nosocomial threat requiring strict adherence to infection control protocols. A confirmatory phenotypic test followed by genotypic tests will help in the correct and accurate identification of this resistance.

## Introduction

Multidrug-resistant (MDR) Gram-negative bacteria are an important public health concern because options of antibiotics available for their treatment are limited. There are many mechanisms by which bacteria can resist the action of beta (β)-lactam antibiotics; one of the primary mechanisms is the production of the AmpC β-lactamase enzyme [1]. In contrast to extended-spectrum β-lactamase (ESBL) producers, ESBL inhibitors (e.g., clavulanic acid) have no impact on the AmpC β-lactamase activity [2].

The Bush-Jacoby-Medeiros functional classification of AmpC enzymes places them in group 1, but the Ambler structural classification places them in class C [3]. AmpC β-lactamase genes were originally transmitted via a chromosomal transfer, but plasmids are also capable of doing so [4]. The plasmid-mediated AmpC (pAmpC) β-lactamases are a serious concern and have caused the emergence and spread of MDR strains, which are important from the clinical and epidemiological standpoints [5].

The first plasmid-encoded AmpC variation was isolated from *Klebsiella pneumoniae* in 1989 from South Korea. Because of its phenotypic characteristic linked to cephamycinase and a well-known cefoxitin resistance, it was given the name CMY-1 [6]. A number of AmpC families have been identified in different parts of the globe. Members of the CMY-1 and CMY-2 families of the CMY β-lactamases are found in *Citrobacter freundii* and* Aeromonas hydrophila*, respectively. Similarly, the ACC family and the LAT family are found in *Hafnia alvei* and *C. freundii,* respectively. The FOX- and MOX-type enzymes originated from the *Aeromonas* spp., the DHA type from* Morganella morganii*, and the MIR and ACT types from* Enterobacter* spp. [7].

The Clinical and Laboratory Standards Institute (CLSI) does not offer standardized screening and confirmation procedures for determining the presence of AmpC, in contrast to carbapenemases and ESBLs. Cefoxitin resistance is used to identify AmpC producers as described in the European Committee on Antimicrobial Susceptibility Testing (EUCAST), although this strategy will not detect pAmpC that do not hydrolyze cefoxitin [8]. Methods for identifying AmpC enzymes are still being developed, although they have not yet been optimized for use in clinical laboratories [9]. For surveillance and hospital infection control, knowing whether or not AmpC β-lactamase enzymes are present and how frequently they appear in clinical isolates may be helpful information. This information will affect the decision on the best antibiotic prescription [10].

We undertook our study to ascertain the prevalence of AmpC β-lactamase and its genotypes among uropathogenic *Escherichia coli* in this geographical area due to the limited literature on the discovery and molecular characterization of the AmpC β-lactamase enzymes in Enterobacteriaceae.

## Materials and methods

This hospital-based study was done from May 2019 to April 2020 in the Microbiology Department of Subharti Medical College, Meerut, India. Prior to the study, the University's Ethics Committee provided a written approval via letter SMC/IEC/2019/93/04. Urine samples received in the laboratory yielded 312 non-repeat clinical *E. coli* isolates. According to the CLSI's 2018 recommendation, antimicrobial susceptibility testing (AST) was carried out by utilizing the Kirby-Bauer disc diffusion method [11].

Phenotypic screening and confirmatory test for AmpC β-lactamase production

Using the disc diffusion method and a cefoxitin (30 µg) disc, the *E. coli *isolates were recognized as the presumptive AmpC β-lactamase producers. Screen positives were defined as an inhibitory zone with the cefoxitin disc surrounding it and a diameter of ≤18 mm [12]. The isolates that were screen positives were subjected to an additional phenotypic test utilizing an inhibitor-based method (IBM). A Mueller-Hinton agar (MHA) plate was inoculated, and then two discs containing 400 µg of boronic acid and 30 µg of cefoxitin each were placed 30 mm apart and incubated overnight. A zone size increase of ≥5 mm in the disc containing cefoxitin and boronic acid over the disc containing cefoxitin alone was deemed positive for the AmpC β-lactamase synthesis [13,14].

Detection of AmpC β-lactamases by polymerase chain reaction (PCR)

The multiplex polymerase chain reaction (PCR) method, which was established by Pérez-Pérez and Hanson [10], was utilized to identify the family-specific plasmid-mediated common AmpC genes, namely, ACC, DHA, CIT, FOX, MOX, and EBC, in the 152 screen-positive isolates. The sequence of the primers used for PCR amplification are listed in (Table 1).

**Table 1:** List of primers for the amplification of different AmpC genes

Target gene	Primers	Sequences (5’ to 3’)	Amplicon size (bp)	References
CMY-8 to CMY-11 MOX-1, MOX-2, CMY-1,	MOXMF MOXMR	Forward: GCTGCTCAAGGAGCACAGGAT Reverse CACATTGACATAGGTGTGGTGC	520	Pérez-Pérez and Hanson [10]
CMY-2 to CMY-7, BIL-1 LAT-1 to LAT-4,	CITMF CITMR	Forward: TGGCCAGAACTGACAGGCAAA Reverse TTTCTCCTGAACGTGGCTGGC	462	Pérez-Pérez and Hanson [10]
ACC	ACCMF ACCMR	Forward AACAGCCTCAGCAGCCGGTTA Reverse TTCGCCGCAATCATCCCTAGC	346	Pérez-Pérez and Hanson [10]
DHA-1, DHA-2	DHAMF DHAMR	Forward: AACTTTCACAGGTGTGCTGGGT Reverse: CCGTACGCATACTGGCTTTGC	405	Pérez-Pérez and Hanson [10]
MIR-1, ACT-1	EBCMF EBCMR	Forward: TCGGTAAAGCCGATGTTGCGG Reverse CTTCCACTGCGGCTGCCAGTT	302	Pérez-Pérez and Hanson [10]
FOX-1 to FOX-5b	FOXMF FOXMR	Forward: AACATGGGGTATCAGGGAGATG Reverse CAAAGCGCGTAACCGGATTGG	190	Pérez-Pérez and Hanson [10]

DNA isolation and purification

The test isolates were obtained as a single colony from a fresh culture plate and inoculated to 5 mL of Luria-Bertani broth and kept at 37 °C for overnight incubation. Following the manufacturer's instructions, DNA was extracted from the samples utilizing the automated DNA extraction tool Trueprep AUTO kit by Molbio Diagnostics Pvt. Ltd. (India) [15].

PCR amplification of genes

A total of 2.5 µL of template DNA; 0.24 µL of forward and reverse MOX, CIT, and DHA primers; 0.20 µL of forward and reverse ACC and EBC primers; 0.16 µL of forward and reverse FOX primers; 12.5 µL of a master mixture (GeNei TM PCR Master Mix, Genei Laboratories Pvt. Ltd., India); and 7.44 µL of DNase/RNase free distilled water were included in the reaction mixture for PCR tests. The initial denaturation was performed for three minutes at 94 °C. This was followed by 25 cycles of DNA denaturation at 94 °C for 30 seconds, primer annealing at 64 °C for 30 seconds, primer extension at 72 °C for one minute, and a final extension step at 72 °C for seven minutes [9].

Gel visualization of the amplified products

The AmpC gene-amplified DNA fragments were visualized using 2.5% agarose gel electrophoresis. Gels were then stained with ethidium bromide at a concentration of 5 µg/mL using a UV transilluminator. As a molecular weight marker, a 100 bp DNA ladder was employed (Genei Laboratories Pvt. Ltd., India). As a negative control, sterile, double-distilled water was added to PCR mixtures in place of the template DNA.

## Results

In our study, 152 (48.72%) of the clinical isolates of *E. coli *tested positive for cefoxitin screen, demonstrating that they generated AmpC. However, the synthesis of AmpC β-lactamase was phenotypically confirmed in 118/152 (77.63%) of the isolates by the IBM. Most isolates that tested positive for AmpC (73%) were from hospitalized patients. The majority of AmpC producing* E. coli* were isolated from patients with the age group of 51-60 years old (29.66%), followed by those between 41 and 50 (25.42%), while those between the age of 11 and 20 (3.38%) had the lowest percentage. There was predominance in female patients aged 65 years (55.08%), and the male:female ratio was 1:1.23 (Table 2).

**Table 2:** Distribution of AmpC-positive isolates among the patients according to age and gender (n=118)

Age Groups	Males n (%)	Females n (%)	Total n (%)
1-10	3 (2.54%)	5 (4.23%)	8 (6.77%)
11-20	2 (1.69%)	2 (1.69%)	4 (3.38%)
21-30	2 (1.69%)	8 (6.77%)	10 (8.47%)
31-40	5 (3.28%)	11 (9.32%)	16 (13.55%)
41-50	11 (9.32%)	19 (16.10%)	30 (25.42%)
51- 60	21 (17.79%)	14 (11.86%)	35 (29.66%)
61-70	5 (4.23%)	4 (3.38%)	9 (7.62%)
>71	4 (3.38%)	2 (1.69%)	6 (5.08%)
Total	53 (44.92%)	65 (55.08%)	118 (100%)

**Figure 1:**
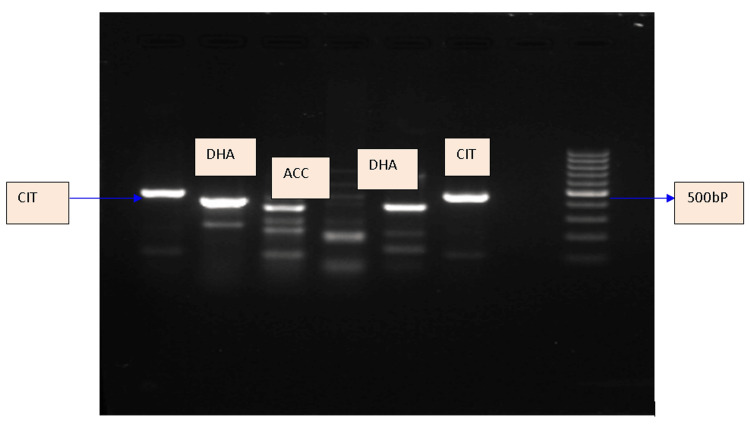
Representative gel image depicting bands of various genes on multiplex PCR. Lane 1 shows CIT (462 bp), lane 2 shows DHA (405 bp), lane 3 shows ACC (346 bp), lane 5 shows DHA (405 bp), lane 6 shows CIT (462 bp), lane 7 shows the negative control, and lane 8 shows the 100 bp ladder.

Out of the 152 screen-positive isolates, genotypes were detected from 56 (36.44%) isolates. The common genotypes detected in this geographical area included CIT, CIT-FOX, DHA, EBC, ACC, FOX, and MOX. The most commonly detected genotype was CIT (57.14%), followed by CIT-FOX (14.29%). Table 3 shows the distribution of AmpC β-lactamase genes among the isolates. Figure 1 shows the representative gel image depicting bands of various genes on multiplex PCR.

**Table 3:** Distribution of AmpC β-lactamase genes among isolates (n=56)

Genes	Number and percentage of AmpC gene-positive isolates	
	n	%
CIT	32	57.14%
CIT-FOX	8	14.29%
EBC	5	8.93%
DHA	5	8.93%
ACC	3	5.36%
FOX	2	3.57%
MOX	1	1.78%
Total	56	100%

The susceptibility patterns of the phenotypically and genotypically confirmed AmpC-positive isolates to various antibiotics are shown in Table 4. The isolates that tested positive for the AmpC enzyme showed complete (100%) resistance toward third-generation cephalosporins, β-lactams, and β-lactamase inhibitor combination and reduced susceptibility toward cefepime, a fourth-generation cephalosporin. However, 100% sensitivity was seen against fosfomycin, and good susceptibility was seen against imipenem (90.68%), followed by nitrofurantoin (81.36%) and amikacin (76.28%). However, based on the chi-square test (χ2: 51.66; with a p-value of 0.00001), there was a significant difference between the results of the phenotypic and genotypic tests (Table 5).

**Table 4:** Resistance patterns of phenotypically and genotypically confirmed AmpC-producing isolates to various antibiotics *p-value statistically significant; N/A: not applicable

Antibiotic used	Phenotypic AmpC-positive isolates (n=118)	Genotypic AmpC-positive isolates (n=56)	p-value
Ampicillin	118 (100%)	56 (100%)	< .00001>
Ceftriaxone	118 (100%)	56 (100%)	< .00001>
Ceftazidime	118 (100%)	56 (100%)	< .00001>
Cefepime	103 (87.29%)	50 (89.28%)	0.70394
Amoxycillin-clavulanic acid	118 (100%)	56 (100%)	< .00001>
Amikacin	28 (23.72%)	18 (32.14%)	0.238
Gentamicin	77 (65.25%)	39 (69.64%)	0.5868
Aztreonam	86 (72.88%)	47 (83.92%)	0.1096
Imipenem	11 (9.32%)	10 (17.85%)	0.1074
Norfloxacin	79 (66.95 %)	44 (78.57%)	0.11642
Nitrofurantoin	22 (18.64 %)	18 (32.14%)	< .0477>
Fosfomycin	0 (0.00%)	0 (0.00%)	N/A

**Table 5:** Comparative evaluation of the phenotypic and genotypic methods for AmpC detection in screen-positive isolates (n=152) IBM: inhibitor-based method; PCR: polymerase chain reaction

Number of AmpC-producing isolates	IBM n(%)	Multiplex PCR n(%)	χ2	p-value
Positive	118 (77.63%)	56 (36.84%)	51.66	<0.00001
Negative	34 (22.37)	96 (63.16%)		
Total	152 (100%)	152 (100%)		

## Discussion

The production of AmpC β-lactamases leads to treatment failure with a broad spectrum of antibiotics [16]. Clinical laboratories lack efficient and precise detection methods, so the understanding of AmpC β-lactamases is currently limited. Studies showed that some bacteria, such as *Klebsiella* and *E. coli*, have a higher propensity to harbor plasmids that encode the AmpC β-lactamase enzyme and frequently exhibit resistance to a variety of antibiotics, including β-lactams [17]. Carbapenems are the preferred treatment for microorganisms that produce AmpC. Meanwhile, non-AmpC producers that are cefoxitin resistant can be treated with extended-spectrum cephalosporins [18].

In our study, 48.72% of *E. coli* were cefoxitin resistant. We observed that 77.63% of the *E. coli *isolates are AmpC β-lactamase positive on the phenotypic screening. Mohamudha et al. [18] discovered AmpC in 63.4% of clinical isolates of *E. coli* and *K. pneumoniae*, which is comparable to the prevalence of our study. However, a Nepalese investigation by Aryal et al. reported a greater frequency of 80.53% than our study [19]. When compared to earlier research studies from the same region, we definitely showed a higher rate of AmpC synthesis [20].

According to findings from India, the Enterobacteriaceae family exhibits a high prevalence of plasmid-mediated AmpC β-lactamases, demonstrating the presence of these enzymes in a significant proportion of these bacterial populations [21]. As the majority of the clinical samples from the in-patient department (IPD) contained isolates of AmpC-producing bacteria (73%), these microorganisms are clearly more frequently acquired or transmitted in a particular healthcare facility.

Among AmpC-producing *E. coli *isolates, 29.66% were isolated from patients with the age group of 51-60 years old, as determined by the demographics of the patients in our study. Shagufta et al. also reported a considerably greater percentage of AmpC producers in the age group of 50-59 years old (37.7%), which is comparable to our study [22]. The elderly are admitted to the hospital at a higher rate than the rest of the age groups and for longer periods, which makes them vulnerable to severe hospital-acquired infections.

The AmpC-positive isolates exhibited resistance toward multiple antimicrobial agents tested along with complete resistance to ampicillin, amoxycillin+clavulanic acid, ceftazidime, and ceftriaxone. The study by Handa et al. [20] found that the isolates of *E. coli *that express the AmpC enzymes are entirely resistant to mixtures of β-lactams, β-lactamase inhibitors, and third-generation cephalosporins, including fourth-generation cephalosporins (cefepime). This underscores the fact that these drugs are no longer appropriate for the empirical therapy of urinary tract infections (UTIs) in this region. The drugs that can be used to treat infections caused by organisms that produce AmpC, such as fosfomycin, imipenem, nitrofurantoin, and amikacin, were nevertheless the most effective ones. Multidrug resistance was much more common among AmpC producers than among AmpC non-producers, pointing to plasmid-mediated dissemination.

We could detect AmpC genes in 56 (36.84%) of the screen-positive isolates. CIT (57.14%) was the most common genotype, followed by CIT-FOX (14.28%), which was the second most common genotype. Another study from Southern India also found that the CIT family, which includes the genes CMY-2 to CMY-7, LAT-1 to LAT-4, and BIL-1, predominates, followed by DHA and EBC [23]. Numerous investigators in India have described various pAmpC genotypes. Chakraborty et al. only found the CIT gene among the isolates in their research [24]. Varghese et al. [25] revealed that the three most prevalent AmpC genotypes are CIT, DHA, and EBC. In contrast to our findings, Govindaswamy et al. [26] found that the most prevalent AmpC genotype among the isolates was the blaFOX gene (21.9%). The majority of the isolates identified by Mohamudha et al. contained plasmid-mediated AmpC genes; the most prevalent molecular subtypes were DHA and CIT types, followed by MOX and ACC types [27]. However, Manoharan et al. identified the primary AmpC subtype as the FOX-CIT gene [28]. Of our isolates, eight (14.28%) had the CIT-FOX gene combination. Nakaye et al. [29] displayed a variety of AmpC subtypes in 26% of the clinical isolates. Due to these geographic variations in the AmpC genes, the development of AmpC subtypes is of special scientific interest globally.

Limitations of the study

Our study has few limitations: i) Due to limited resources, gene sequencing could not be performed for the AmpC genes detected in* E. coli*. ii) Instead of focusing on finding the existence of plasmid-mediated AmpC β-lactamase as the likely mechanism, the alternative potential mechanisms for cefoxitin resistance were not investigated. iii) We did not look for additional resistance mechanisms, including the production of ESBLs, efflux pumps, and metallo-β-lactamses (MBLs). iv) As this study was performed in a particular hospital with a limited geographical area and since isolates from one hospital cannot entirely cover the geographical area, the findings cannot be generalized to the entire population.

## Conclusions

AmpC β-lactamase-producing strains in the current study exhibited a significant MDR level and were resistant to the majority of existing antibiotics. Effective therapeutic intervention requires knowledge of the prevalence and molecular subtypes of plasmid-mediated AmpC β-lactamases. Furthermore, it is necessary to conduct studies on a large scale with more representative samples that can evaluate the prevalence of AmpC β-lactamase producers from a wider geographical perspective. Many of the cefoxitin-resistant strains in this investigation tested negative by PCR for AmpC enzymes, demonstrating the existence of additional mechanisms of resistance. This study discovered an alarmingly high incidence of AmpC β-lactamase in uropathogenic *E. coli* in this region, which calls for ongoing surveillance to halt the spread of these strains in hospitals and improve infection control protocols.

## References

[1] Najjuka CF, Kateete DP, Lodiongo DK (2020). Prevalence of plasmid-mediated AmpC beta-lactamases in Enterobacteria isolated from urban and rural folks in Uganda. AAS Open Res.

[2] Bush K, Jacoby GA (2010). Updated functional classification of beta-lactamases. Antimicrob Agents Chemother.

[3] Jacoby GA (2009). AmpC beta-lactamases. Clin Microbiol Rev.

[4] Santiago GS, Gonçalves D, da Silva Coelho I, de Mattos de Oliveira Coelho S, Neto Ferreira H (2020). Conjugative plasmidic AmpC detected in Escherichia coli, Proteus mirabilis and Klebsiella pneumoniae human clinical isolates from Portugal. Braz J Microbiol.

[5] Saffar H, Niaraki AN, Tali AG, Baseri Z, Abdollahi A, Yalfani R (2016). Prevalence of AMPC β-lactamase in clinical isolates of escherichia coli, Klebsiella spp., and proteus mirabilis in a tertiary hospital in Tehran, Iran. Jundishapur J Microbiol.

[6] Philippon A, Arlet G, Jacoby GA (2002). Plasmid-determined AmpC-type beta-lactamases. Antimicrob Agents Chemother.

[7] Alvarez M, Tran JH, Chow N, Jacoby GA (2004). Epidemiology of conjugative plasmid-mediated AmpC beta-lactamases in the United States. Antimicrob Agents Chemother.

[8] (2023). EUCAST guidelines for detection of resistance mechanisms and specific resistances of clinical and/or epidemiological importance. https://www.eucast.org/fileadmin/src/media/PDFs/EUCAST_files/Resistance_mechanisms/EUCAST_detection_of_resistance_mechanisms_170711.pdf.

[9] Gupta G, Tak V, Mathur P (2014). Detection of AmpC β lactamases in gram-negative bacteria. J Lab Physicians.

[10] Pérez-Pérez FJ, Hanson ND (2002). Detection of plasmid-mediated AmpC beta-lactamase genes in clinical isolates by using multiplex PCR. J Clin Microbiol.

[11] Wayne PA (2018). Clinical and Laboratory Standards Institute: performance standards for antimicrobial susceptibility testing: 20th informational supplement. Clinical and Laboratory Standards Institute.

[12] Ratna AK, Menon I, Kapur I, Kulkarni R (2003). Occurrence &amp; detection of AmpC beta-lactamases at a referral hospital in Karnataka. Indian J Med Res.

[13] Coudron PE (2005). Inhibitor-based methods for detection of plasmid-mediated AmpC beta-lactamases in Klebsiella spp., Escherichia coli, and Proteus mirabilis. J Clin Microbiol.

[14] Hemalatha V, Padma M, Sekar U, Vinodh TM, Arunkumar AS (2007). Detection of Amp C beta lactamases production in Escherichia coli & Klebsiella by an inhibitor based method. Indian J Med Res.

[15] (2023). DNA extraction kit (Trueprep AUTO) Molbio Diagnostics Pvt. Ltd. https://www.molbiodiagnostics.com/product_details.php?id=47.

[16] Doi Y, Paterson DL (2007). Detection of plasmid-mediated class C beta-lactamases. Int J Infect Dis.

[17] Woodford N, Reddy S, Fagan EJ (2007). Wide geographic spread of diverse acquired AmpC beta-lactamases among Escherichia coli and Klebsiella spp. in the UK and Ireland. J Antimicrob Chemother.

[18] Mohamudha PR, Harish BN, Parija SC (2012). Molecular description of plasmid-mediated AmpC β-lactamases among nosocomial isolates of Escherichia coli &amp; Klebsiella pneumoniae from six different hospitals in India. Indian J Med Res.

[19] Aryal SC, Upreti MK, Sah AK (2020). Plasmid-mediated AmpC β-Lactamase CITM and DHAM genes among gram-negative clinical isolates. Infect Drug Resist.

[20] Handa D, Pandey A, Asthana AK, Rawat A, Handa S, Thakuria B (2013). Evaluation of phenotypic tests for the detection of AmpC beta-lactamase in clinical isolates of Escherichia coli. Indian J Pathol Microbiol.

[21] Kazi M, Ajbani K, Tornheim JA, Shetty A, Rodrigues C (2019). Multiplex PCR to detect pAmpC β-lactamases among enterobacteriaceae at a tertiary care laboratory in Mumbai, India. Microbiology (Reading).

[22] Shagufta R, Fomda B, Gulnaz B, Lubna S, Jan A, Mohd S, Junaid A (2017). Prevalence of AMPC beta-lactamase in gram negative bacilli by different phenotypic methods in a tertiary care institute in Kashmir. J Adv Med Med Res.

[23] Shanthi M, Sekar U, Arunagiri K, Sekar B (2012). Detection of Amp C genes encoding for beta-lactamases in Escherichia coli and Klebsiella pneumoniae. Indian J Med Microbiol.

[24] Chakraborty A, Adhikari P, Shenoy S, Saralaya V (2014). Characterization of plasmid mediated AmpC producing Escherichia coli clinical isolates from a tertiary care hospital in South India. Indian J Pathol Microbiol.

[25] Varghese DS, Sekar U, Shanthi M, Arunagiri K, Vishwanathan A, Vidhya VM, Sekar B (2014). Concurrent occurrence of Amp C and Cefotaxime (CTX)-M in clinical isolates of enterobacteriaceae. J Acad Clin Microbiol.

[26] Govindaswamy A, Bajpai V, Khurana S, Aravinda A, Batra P, Malhotra R, Mathur P (2019). Prevalence and characterization of beta-lactamase-producing Escherichia coli isolates from a tertiary care hospital in India. J Lab Physicians.

[27] Mohamudha Parveen R, Harish BN, Parija SC (2010). Ampc beta lactamases among gram negative clinical isolates from a tertiary hospital, South India. Braz J Microbiol.

[28] Manoharan A, Sugumar M, Kumar A, Jose H, Mathai D (2012). Phenotypic & molecular characterization of AmpC β-lactamases among Escherichia coli, Klebsiella spp. &amp; Enterobacter spp. from five Indian medical centers. Indian J Med Res.

[29] Nakaye M, Bwanga F, Itabangi H, Stanley IJ, Bashir M, Bazira J (2014). AmpC-BETA lactamases among Enterobacteriaceae isolated at a tertiary hospital, South Western Uganda. Br Biotechnol J.

